# Antibody-based binding domain fused to TCRγ chain facilitates T cell cytotoxicity for potent anti-tumor response

**DOI:** 10.1038/s41389-023-00480-4

**Published:** 2023-06-22

**Authors:** Zhao Chen, Changyou Lin, Hong Pei, Xiaomei Yuan, Jia Xu, Mingwei Zou, Xinyuan Zhang, Amber Fossier, Meizhu Liu, Seungah Goo, Lei Lei, Jia Yang, Catherine Novick, Jiqing Xu, Ge Ying, Zhihong Zhou, Jianbo Wu, Chunyi Tang, Wenying Zhang, Zhenping Wang, Zhihao Wang, Huitang Zhang, Wenzhong Guo, Qidong Hu, Henry Ji, Runqiang Chen

**Affiliations:** Sorrento Therapeutics Inc, 4955 Directors Place, San Diego, CA USA

**Keywords:** Immunotherapy, Cancer immunotherapy

## Abstract

Chimeric antigen receptor T-cell (CAR-T) therapy has demonstrated potent clinical efficacy in the treatment of hematopoietic malignancies. However, the application of CAR-T in solid tumors has been limited due in part to the expression of inhibitory molecules in the tumor microenvironment, leading to T-cell exhaustion. To overcome this limitation, we have developed a synthetic T-cell receptor (TCR) that targets programmed death-ligand 1 (PD-L1), a molecule that is widely expressed in various solid tumors and plays a pivotal role in T-cell exhaustion. Our novel TCR platform is based on antibody-based binding domain, which is typically a single-chain variable fragment (scFv), fused to the γδ TCRs (TCRγδ). We have utilized the T-cell receptor alpha constant (TRAC) locus editing approach to express cell surface scFv of anti-PD-L1, which is fused to the constant region of the TCRγ or TCRδ chain in activated T cells derived from peripheral blood mononuclear cells (PBMCs). Our results indicate that these reconfigured receptors, both γ-TCRγδ and δ-TCRγδ, have the capability to transduce signals, produce inflammatory cytokines, degranulate and exert tumor killing activity upon engagement with PD-L1 antigen in vitro. Additionally, we have also shown that γ-TCRγδ exerted superior efficacy than δ-TCRγδ in in vivo xenograft model.

## Introduction

Engineered T cells have revolutionized the field of cancer therapy, offering a new approach for targeting and destroying cancer cells. T-cell receptor-engineered T-cell therapy (TCR-T) and chimeric antigen receptor-engineered T-cell therapy (CAR-T) are two promising adoptive cell therapy (ACT) approaches for cancer treatment [1, 2]. The prototypical CAR-T design features a synthetic receptor that expresses antigen binding domains in a single chain format fused to the immunoreceptor tyrosine-based activation motif (ITAM) containing domains of CD3ζ, thus generating a synthetic means of activating CAR-expressing cells [3]. To achieve enhanced persistence and tumor killing activity, the second and third generations of CAR-T further incorporated co-stimulatory domains such as those derived from host cell proteins CD28 and 4-1BB [4]. Over the decades, CAR-T has been proved to be highly effective in treating hematopoietic cancers [5–8], with a total of five CD19 CAR-T and two BCMA CAR-T therapies having been approved by the U.S. Food and Drug Administration (FDA) for the treatment of certain hematopoietic malignancies. These approvals represent significant progress in the development and commercialization of CAR-T cell therapy and offer new hope for patients with these types of cancers. Nevertheless, much less has been achieved in the treatment of solid tumors, largely due to the heterogeneity of tumor antigens, poor tumor-infiltration, and T cell exhaustion in solid tumors [9–13]. While CAR-T binds the surface antigen in an MHC-independent manner, TCR-T can recognize many other types of antigens presented in the context of a peptide-MHC complex and propagate the signals via the endogenous TCR-CD3 complex [14, 15]. Moreover, TCR-T has structural advantages which result in higher antigen sensitivity and better anti-tumor efficacy than CAR-T in the solid tumor settings [14, 15]. However, the difficulties in selecting tumor-specific targets and screening for optimal TCR affinity to a given target limit the broader application of TCR-based cell therapy in patients.

Given the distinct features of CAR-T and TCR-T, great efforts have been made to develop novel hybrid platforms to harness the strengths of both strategies. Candidate platforms have been generated by conjugating the extracellular portion of CAR-T to the cytoplasmic domain of TCR, or, alternatively, by ligating the extracellular MHC-peptide recognition portion of TCR to the cytoplasmic domain of CAR-T [16–20]. One example is the antibody-TCR (AbTCR) which constitutes a fusion of the antibody fragment antigen-binding domain (Fab) to the endogenous TCR-CD3 complex [18, 20]. In such a way, the binding of the Fab to a tumor antigen can trigger TCR activation through the natural TCR. Previous studies have shown that both TCRαβ chains and γδ chains can form TCR-CD3 complexes to propagate the cellular activation signals and exert subsequent anti-tumor activity [20, 21]. Another study reported the fusion of CD19 scFv to CD3 subunits could utilize antigen-specific activation of TCR signaling, while scFv-CD3ε engineered T cells showed better anti-tumor efficacy than CAR-T [16].

Programmed death-ligand PD-L1 is abundantly expressed in many tumors, which makes it a potential universal tumor antigen targeted by an engineered T cell [22, 23]. Moreover, the binding of PD-L1 to PD1, an immune checkpoint molecule expressed on effector T cells, is one of the main mechanisms leading to T cell exhaustion and consequently tumor evasion from the adaptive immunity [24]. PD-L1 expression on T cells upon TCR/CD3 activation converts the cells to a highly suppressive phenotype and could play important roles in maintaining a tolerogenic environment [25]. Therefore, targeting PD-L1 could not only specifically lift the immune suppression caused by tumor cells but may also have potential to enhance the anti-tumor immunity. Indeed, the blockade of PD1/PD-L1 by administering anti-PD1, or anti-PD-L1, has been demonstrated to be effective in patients with non-small cell lung cancer (NSCLC) [26, 27]. Likewise, several studies have reported that targeting PD1/PD-L1 by engineering either anti-PD-L1 scFv CAR or dominant-negative PD1 into T cells suppresses tumor growth [28, 29].

In this study, we designed a new antigen binding receptor structure by fusing an antibody-based binding domain (scFv) targeting PD-L1 to the constant regions of either the γ or δ chain of TCRγδ. These reconfigured receptors, both γ-TCRγδ and δ-TCRγδ, are capable of transducing signals, producing inflammatory cytokines, degranulating and exerting tumor killing activity upon engagement with PD-L1 antigen in vitro. Potent antitumor activities were also recorded in multiple xenograft models of solid tumors. For better comparison with traditional CAR-T design, we applied and validated this novel receptor design with the well-studied CD19 target. Our data suggests that this new platform of scFv-γ-TCRγδ has the potential to greatly advance the field of cancer immunotherapy and lead to better treatments for patients with cancers.

## Results

### Design and characterization of anti-PD-L1 TCRγδ-T cells

**Fig. 1:**
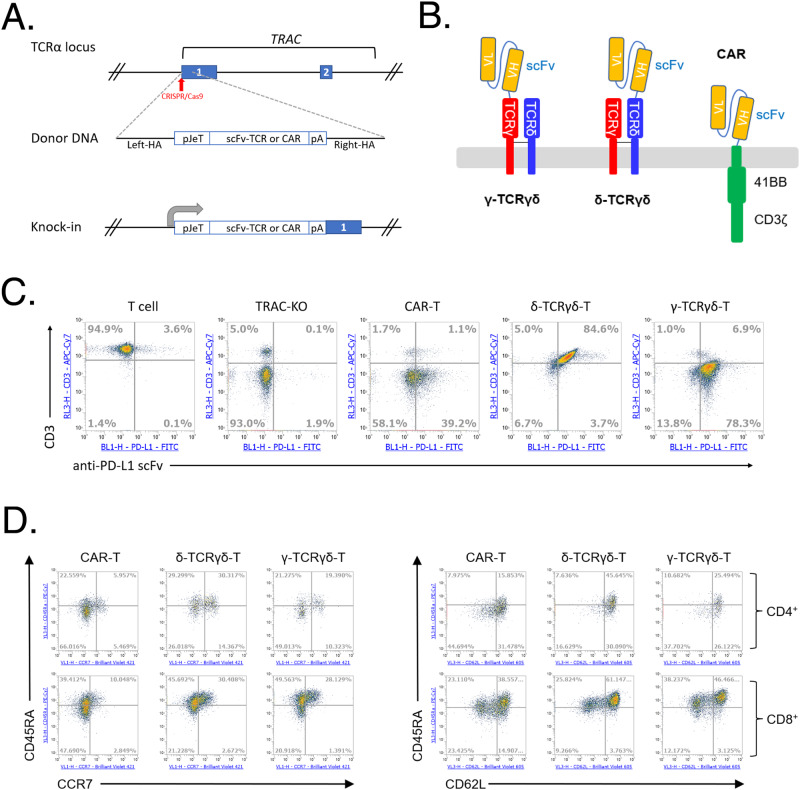
The synthetic anti-PD-L1 TCRγδ design and expression. **A** Illustration of CRISPR/Cas9 mediated knockout and knock-in strategy. The numbered boxes indicate exons of *TRAC*. **B** Schematic diagram of the γ-TCRγδ, δ-TCRγδ compared to a second-generation 41BB-CAR. **C** Flow cytometry result of CAR-T, δ-TCRγδ-T or γ-TCRγδ-T cells. The expression of anti-PD-L1 scFv was detected by staining with PD-L1-Fc. The expression of CD3ε was also assessed. Shown are representative of T cells with CD3ε and anti-PD-L1 scFv expression on day 10 after transfection. **D** T cell memory markers of anti-PD-L1 CAR-T, δ-TCRγδ-T or γ-TCRγδ-T cells. The expressions of CD45RA, CCR7, and CD62L were examined by gating anti-PD-L1 scFv expressing CD4^+^ or CD8^+^ T cells. Shown are representative CD45RA, CCR7, and CD62L expression on anti-PDL1^+^ CD4^+^or anti-PDL1^+^ CD8^+^ T cells at day 13 after transfection.

To overexpress the synthetic antigen binding receptor, we applied CRISPR/Cas9 mediated knockout and knock-in methodology to target the *TRAC* locus in primary T cells isolated from healthy human peripheral blood (PBMC) [30–32] (Fig. 1A). We fused the scFv to the TCRγδ to minimize the interference from endogenous TCRβ chain after knockout of TCRα: the original antigen-binding domain of heterodimeric TCRγδ was replaced by the anti-PD-L1 scFv which was fused to the constant domain of either the δ chain (TCRδ) or the γ chain (TCRγ), with the other constant chain co-expressed via the T2A self-cleavage peptide within the same open reading frame. The resultant receptors were designated as δ-TCRγδ or γ-TCRγδ, respectively, and the expression of transgene was driven under a synthetic pJeT promoter. As control, a second-generation CAR was constructed by fusing the anti-PD-L1 scFv to the scaffold composed of CD8α/CD28 hinge and transmembrane domain (TMD), 4-1BB costimulatory domain and CD3ζ signaling domain (Fig. 1B and Supplementary Table 1).

We first validated by flow cytometry that the anti-PD-L1 scFv was efficiently expressed on the surface of both δ-TCRγδ-, γ-TCRγδ- and CAR-T cells (Fig. 1C). While the CAR-T and TRAC-KO cells showed negative CD3ε expression on the surface as the expected result of TCRα knockout, the CD3ε was detected on the surface of the δ-TCRγδ-T cells, suggesting that the scFv fused to δ-TCRγδ may still assemble with the CD3 components including CD3γ, δ, ε, and ζ subunits, thus restored the CD3ε surface expression to the similar level as the non-targeted T cells. However, when the scFv fused to γ-TCRγδ, the cells showed reduced CD3ε expression level compared to the δ-TCRγδ-T and non-targeted T cells. This difference in CD3ε expression level could be attributed to the dissimilarity between the TCRδ and TCRγ chains in terms of their stoichiometry with CD3 subunits [33]. Another difference we observed is that the percentage of scFv^+^ population of CAR-T was lower than either δ-TCRγδ or γ-TCRγδ-T. Despite the difference in CD3ε and scFv expression, both the δ-TCRγδ-T and γ-TCRγδ-T cells exhibited similar phenotype regarding the memory marker expression, in comparison with CAR-T cells (Fig. 1D).

### TCRγδ-T cells exhibited robust effector functions against various tumor cell lines

**Fig. 2:**
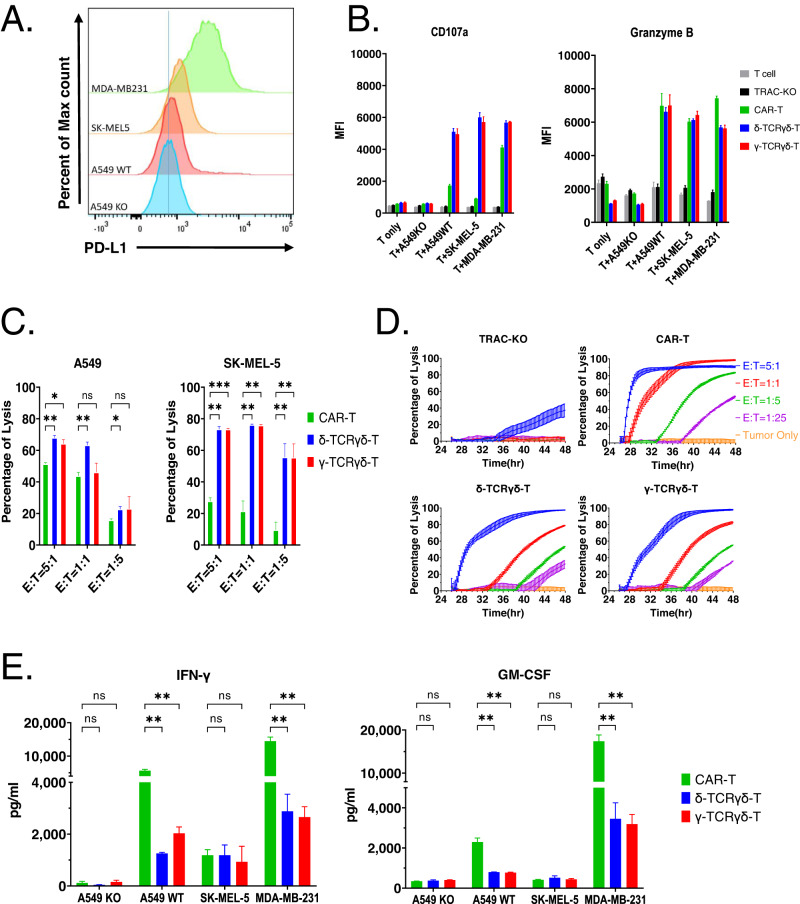
The synthetic anti-PD-L1 TCRγδ function against tumor cell lines. **A** PD-L1 antigen expression in tumor cell lines used in this study was detected with flow cytometry. The histogram displays the overlay of PD-L1-stained tumor cells. **B** Expression of degranulation markers CD107a and Granzyme B. The scFv expressing T cells were cultured alone, or cocultured with A549, A549KO, SK-MEL-5 or MDA-MB-231 in the presence of Brefeldin A. The expression of CD107a and granzyme B was examined by FACS. Mean fluorescence intensity (MFI) of CD107a and granzyme B expression gated on CD8^+^ cells were presented. **C** Representative cytotoxic activity of CAR-T, δ-TCRγδ-T or γ-TCRγδ-T cells towards A549 and SK-MEL-5 cells. The scFv expressing T cells at indicated E/T ratios were added to the tumor cell culture. Cytotoxicity was examined after 4 h of co-culturing by staining tumor cells with Fixable viability dye and Annexin V. Percent of lysis was calculated by normalization to tumor only samples. **D** Representative cytotoxic activity of CAR-T, δ-TCRγδ-T or γ-TCRγδ-T cells towards MDA-MB-231 cells. The scFv expressing T cells at indicated E/T ratios were added to the tumor cell culture. Cytotoxicity was measured with xCELLigence over a 48 h period. Percent of lysis was calculated by normalization to tumor only samples at each time point. **E** Secreted cytokine levels by CAR-T, δ-TCRγδ-T or γ-TCRγδ-T cells. CAR, δ-TCRγδ or γ-TCRγδ expressing T cells were incubated with A549, A549KO, SK-MEL-5 or MDA-MB-231 cells at 1:1 ratio. Supernatants were collected after overnight incubation. The cytokine levels were measured by ELISA. All representative data shown are from 2 different donors and experiments were repeated using at least 2 different donors. All data are mean ± SEM. *p < 0.05, **p < 0.01, ***p < 0.001, ****p < 0.0001, ns: not significant.

We used A549 lung carcinoma cell line and A549-KO (PD-L1 knockout), SK-MEL-5 melanoma cell line and MDA-MB-231 breast cancer cell line to test the tumor killing function of TCRγδ-T cells compared to CAR-T cells. These tumor cell lines vary at the PD-L1 antigen expression level with A549 having the lowest and MDA-MB-231 having the highest compared to A549-KO (Fig. 2A). The same number of PD-L1-scFv^+^ T cells from the three groups of engineered T cells were stimulated at equal ratio with A549, SK-MEL-5 or MDA-MB-231 cells. The degranulation markers CD107a and Granzyme B were analyzed on the CD8+ cytotoxic T subpopulation after co-culturing with the tumor cells. Remarkably, CAR-T, δ-TCRγδ-T and γ-TCRγδ-T cells showed significantly higher levels of degranulation, reflected by increased expression of CD107a and granzyme B, as compared to TRAC-KO cells or non-targeted T cells. On the other hand, when co-cultured with the PD-L1-knockout A549 cells, minimal elevation of granzyme B or CD107 expression was observed, suggesting that the activation of the three types of engineered T cells was triggered by the targeted antigen (Fig. 2B and Supplementary Fig. 1).

Consistently, the three types of synthetic T cells demonstrated efficient killing of the A549, SK-MEL-5 and MDA-MB-231 tumor cells in a dose-dependent manner, with the strongest tumor killing activity recorded at the highest effector/target (E/T) ratio of 5:1 (Fig. 2C, D). We noticed that the killing ability among CAR-T, δ-TCRγδ-T and γ-TCRγδ-T cells is likely correlated with PD-L1 expression in different tumor lines. With PD-L1 low expressing A549 and SK-MEL-5 cells, δ-TCRγδ-T and γ-TCRγδ-T cells significantly outperformed CAR-T cells at the 5:1 E/T ratio, while CAR-T cells outperformed δ-TCRγδ-T and γ-TCRγδ-T cells against PD-L1 high expressing MDA-MB-231 cells at all E/T ratios tested.

The variation of tumor killing effect was also reflected by the inflammatory cytokines IFN-γ and GM-CSF produced after overnight killing of tumor cells. Surprisingly, when co-cultured with tumor cells, the level of cytokine released by δ-TCRγδ-T and γ-TCRγδ-T cells, such as IFNγ and GM-CSF, was lower than those by CAR-T cells (Fig. 2E), except that in SK-MEL-5 cells, which could account for the ineffective killing of SK-MEL-5 by CAR-T cells (Fig. 2C). This observation is consistent with the previous finding that Fab reconfigured γδ TCR-T cells produced less cytokines due to the higher sensitivity of TCR to antigen [20]. Taken together, these results indicate that the reconfigured δ-TCRγδ-T and γ-TCRγδ-T cells are functional and possess potent tumor killing activity in vitro.

### TCRγδ-T cells eradicated PD-L1 tumors in vivo

**Fig. 3:**
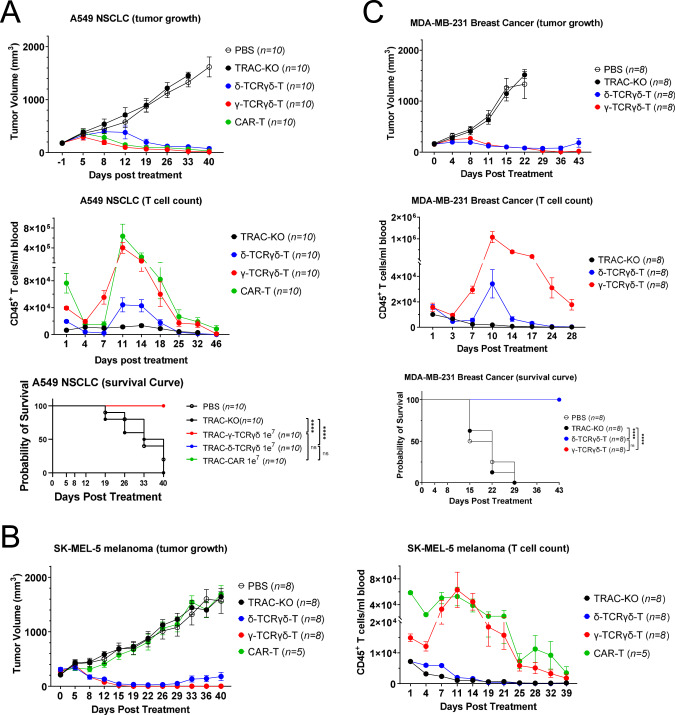
γ-TCRγδ-T cells display superior anti-tumor efficacy in vivo. **A** A549 WT-bearing NSG mice were treated with 1e7 CAR-T (*n* = 10) or δ-TCRγδ T (*n* = 10) or γ-TCRγδ T cells (*n* = 10), as well as equal number of TRAC-KO T cells as a control group (*n* = 10). Tumor size (left panel) was measured at indicated time points and analyzed over a 46-day period. Peripheral blood was drawn at indicated time points and numbers of CD45^+^ cells were calculated (middle panel). Kaplan-Meier survival curve (right panel) was shown with statistical significance calculated using the log-rank Mantel-Cox test (*n* = 10 per cohort). **B** SK-MEL-5 bearing NSG mice were infused with 1e7 TRAC-KO (*n* = 8), CAR-T (*n* = 5), δ-TCRγδ T (*n* = 8) or γ-TCRγδ T cells (*n* = 8). Tumor size (left panel) was measured at indicated time points and analyzed over a 40-day period. Peripheral blood was drawn at indicated time points and numbers of CD45^+^ cells were calculated (middle panel). Kaplan-Meier survival curve (right panel) was shown with statistical significance calculated using the log-rank Mantel-Cox test (*n* = 8 per cohort). **C** MDA-MB-231 bearing NSG mice received 1e7 TRAC-KO (*n* = 8), δ-TCRγδ T (*n* = 8) or γ-TCRγδ T cells (*n* = 8). Tumor size (left panel) was measured at indicated time points and analyzed over a 40-day period. Peripheral blood was drawn at indicated time points and numbers of CD45^+^ cells were calculated (middle panel). Kaplan-Meier survival curve (right panel) was shown with statistical significance calculated using the log-rank Mantel-Cox test (*n* = 8 per cohort). All experiments were repeated using T cells from 2 different donors. All data are mean ± SEM. *p < 0.05, **p < 0.01, ***p < 0.001, ****p < 0.0001, ns: not significant.

We further investigated whether these TCRγδ-T cells would show robust anti-tumor activity in vivo. In a NSCLC xenograft model, treatment with the anti-PD-L1 CAR-T, δ-TCRγδ-T or γ-TCRγδ-T cells generated from two different donors effectively suppressed tumor progression for up to 46 days after infusion (Fig. 3A and Supplementary Fig. 2). Additionally, to characterize the expansion of T cells in vivo, we analyzed the CD45^+^ population in the peripheral blood drawn from the treated mice. Flow cytometry data revealed a significant increase in CD45^+^ T cells from day 7 after adoptive CAR-T cell transfer, followed by a steady decline from day 11 to day 46, indicating a robust T cell expansion upon the encounter of tumor antigen and the subsequent contraction of T cells when tumor was cleared. In contrast to the CAR-T group, δ-TCRγδ-T cells exhibited only limited expansion in vivo, while γ-TCRγδ-T showed comparable expansion level as the CAR-T cells (Fig. 3A and Supplementary Fig. 2). All the 10 mice in each of the CAR-T, δ-TCRγδ-T and γ-TCRγδ-T groups from one donor survived to the end of study (Fig. 3A). With T cells from another donor, 9 out of 10 mice from each of the γ-TCRγδ-T and δ-TCRγδ-T groups, and 10 out of 10 mice from CAR-T group survived to the end of the study (Supplementary Fig. 2). Using cells derived from two donors, we consistently noticed that the δ-TCRγδ-T group showed less efficacy in suppressing the tumor growth compared to γ-TCRγδ-T and CAR-T group in A549 xenograft model (Fig. 3A and Supplementary Fig. 2).

To explore whether TCRγδ-T can also eliminate PD-L1-expressing tumor cells in other cancer types, we tested the efficacy of CAR-, δ-TCRγδ-T or γ-TCRγδ-T cells in the SK-MEL-5 melanoma xenograft model. Similarly, while tumor grew rapidly in mice treated with PBS or TRAC-KO controls, both δ-TCRγδ-T and γ-TCRγδ-T cells effectively repressed the tumor growth up to 40 days after infusion; again the CAR-T cells failed to control the tumor growth despite that a high number of CAR-T cells were detected in the peripheral blood (Fig. 3B). This lack of efficacy of CAR-T vs SK-MEL-5 xenograft may be explained by the ineffective killing of SK-MEL-5 in vitro by the same batch of CAR-T cells (Fig. 2C). It’s worth noting that the γ-TCRγδ-T cells showed greater T cell expansion compared to δ-TCRγδ-T cells, although the latter also managed to control the tumor growth in this xenograft model.

Moreover, in another MDA-MB-231 breast cancer xenograft model, both δ-TCRγδ-T and γ-TCRγδ-T cells were able to suppress the tumor growth up to 36 days, before the tumor relapsed in the δ-TCRγδ-T group (Fig. 3C). Consistent with other xenograft models, the γ-TCRγδ-T cells still showed better expansion than δ-TCRγδ-T cells (Fig. 3C). All the mice in each of the CAR-T, δ-TCRγδ-T and γ-TCRγδ-T groups survived to the end of study, where all mice from the PBS and TRAC-KO group died before day 29 (Fig. 3C). Taken together, the anti-PD-L1 scFv fused to TCRγδ showed superior anti-tumor efficacy in three xenograft models.

### ScFv fused to the γ-TCRγδ demonstrated stronger anti-tumor efficacy than δ-TCRγδ

**Fig. 4:**
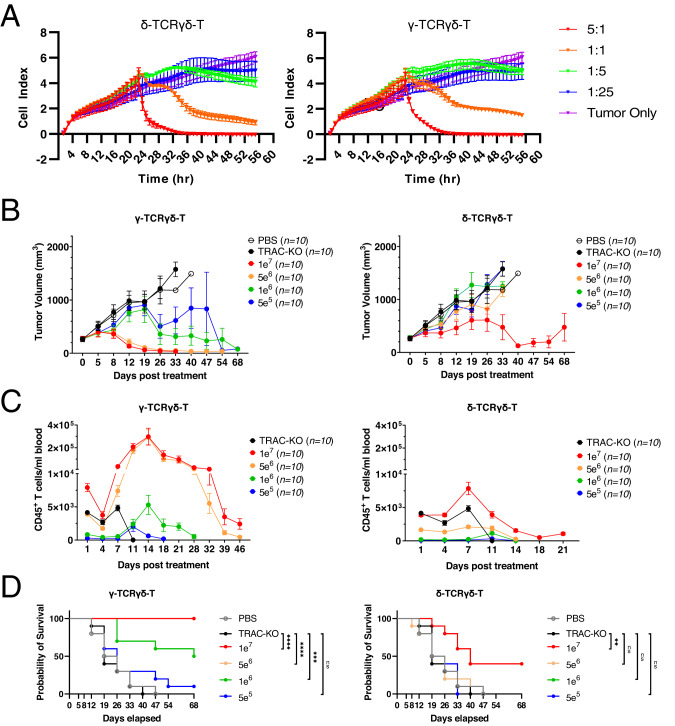
γ-TCRγδ-T and δ-TCRγδ-T dosing comparison in vitro and in vivo. **A** Representative cytotoxic activity of δ-TCRγδ-T or γ-TCRγδ-T cells as measured by tumor cell growth/adherence. One day before the addition of effector T cells, A549 cells were seeded. After adding the effector cells at indicated E/T ratio, the real time measurement of T cell mediated cytolysis was taken with xCELLigence over a 65 h period. **B** A549 WT-bearing NSG mice were treated with different numbers of δ-TCRγδ-T (*n* = 10) or γ-TCRγδ-T cells (*n* = 10), as well as equal number of TRAC-KO T cells (*n* = 10) as a control group. Tumor size was measured at indicated time points and analyzed over a 68-day period. **C** Peripheral blood was analyzed at indicated time points and numbers of CD45^+^ cells were calculated. **D** Kaplan-Meier survival curve was shown with statistical significance calculated using the log-rank Mantel-Cox test (*n* = 10 per cohort). All data are mean ± SEM. *p < 0.05, **p < 0.01, ***p < 0.001, ****p < 0.0001, ns: not significant.

Given the dissimilarity between TCRδ and γ chains in terms of their stoichiometry with CD3 subunits [33], the variations in antitumor efficacy and T cell expansion ability between δ-TCRγδ-T and γ-TCRγδ-T cells encouraged us to further explore their antitumor activity in a dose dependent manner in the A549 NSCLC xenograft model, in which a slight difference of anti-tumor efficacy was observed in the previous single dosing study (Fig. 3A). We first compared the in vitro killing ability of δ-TCRγδ-T and γ-TCRγδ-T cells at different E/T ratio using RTCA but did not observe a significant difference at lysing the A549 cells (Fig. 4A). We then administered various numbers of scFv^+^ γ-TCRγδ-T or δ-TCRγδ-T cells into the mice after tumor engraftment and tracked the tumor growth in a longer period up to 68 days post infusion of T cells. We observed that γ-TCRγδ-T cells exhibited a dose-dependent suppression of tumor growth, with tumor eradication by the higher doses of 1 × 10^7^ and 5 × 10^6^ γ-TCRγδ-T cells (Fig. 4B). In contrast, none of the doses tested for δ-TCRγδ-T could completely inhibit the tumor progression. In fact, although the highest dose of 1 × 10^7^ δ-TCRγδ-T cells suppressed the tumor volume close to the baseline between day 40 to 54, eventually the tumor volume began to grow (Fig. 4B). Correspondingly, greater T cell expansion in peripheral blood was detected in mice infused with γ-TCRγδ-T cells, especially at the two highest doses, 1 × 10^7^ and 5 × 10^6^, which could account for the superior control of the tumor size (Fig. 4C). We also observed a significantly higher survival rate in the lower dose 1e^6^ of γ-TCRγδ-T group compared to TRAC-KO group, while only the highest dose 1e^7^ of δ-TCRγδ-T group had significant survival rate. These findings demonstrated the potent anti-tumor property of γ-TCRγδ-T cells and revealed the differences between the δ-TCRγδ- and γ-TCRγδ-T cells, albeit their comparable in vitro tumor killing activity.

### γ-TCRγδ-T cells effectively infiltrated to the tumor site and persisted in vivo

**Fig. 5:**
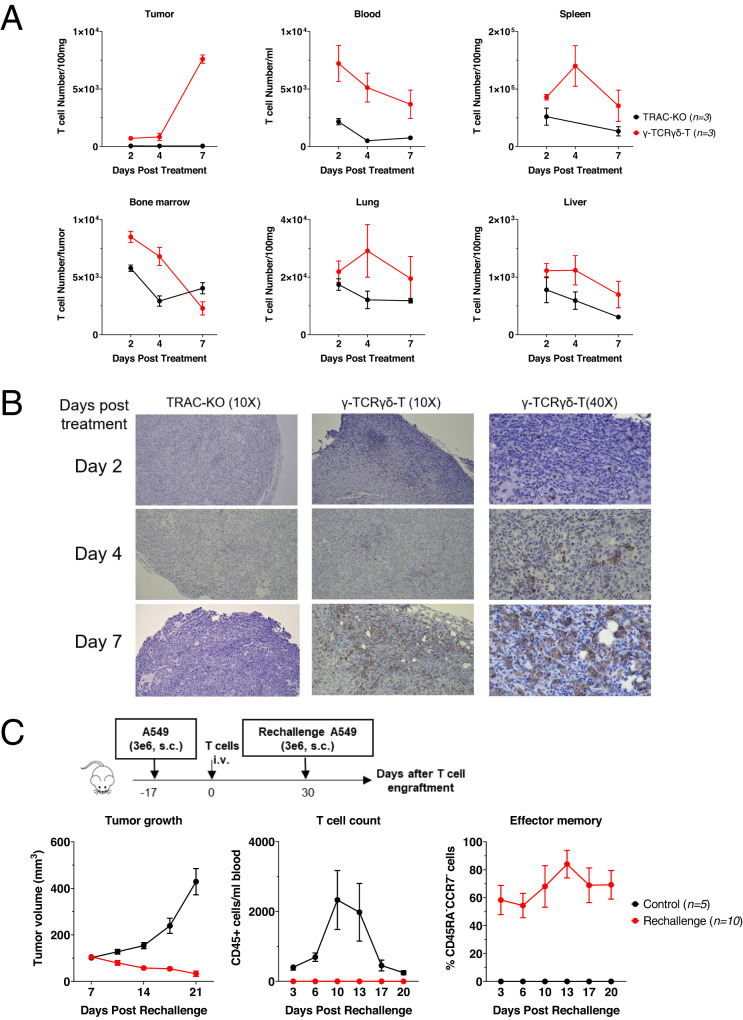
γ-TCRγδ-T cells effectively infiltrate to the tumor site and persist in vivo. **A** A549 WT-bearing NSG mice were treated with 1e7 of γ-TCRγδ-T cells (*n* = 3) or TRAC-KO T cells (*n* = 3) as control. On indicated days, subcutaneous tumors were removed to analyze the T cells within the tumors. Numbers of CD45^+^ T cells in the tumor site were evaluated by flow cytometry. **B** Representative immunohistochemistry (IHC) staining of CD45^+^ T cells within the tumors. The brown-stained cells represent human CD45-positive T cells. **C** Schematic diagram of rechallenge of A549 WT cells in NSG mice. A549 WT-bearing NSG mice were initially treated with 1e7 γ-TCRγδ-T cells (*n* = 10). After tumor elimination, mice were rechallenged with A549 WT cells, tumor size was measured at indicated time points after rechallenging and analyzed over a 21-day period. Peripheral blood was drawn at indicated time points and numbers of CD45^+^ cells were calculated. Percentage of effector memory population, CD45RA-CCR7- T cells, was shown.

Cell trafficking further revealed that γ-TCRγδ-T cells successfully infiltrated into the tumor site 7 days after infusion, which coincided with the shrinkage of tumor lump starting from day 8 post treatment. In contrast, TRAC-KO cells failed to infiltrate into the tumor (Fig. 5A, B). We then asked whether T cells could persist after tumor eradication and proliferate again when re-encountering the same tumor antigen. Hence, upon the elimination of A549 tumor xenograft, the mice were re-challenged with the same tumor cells. Remarkably, the CD45^+^ cells underwent a rapid surge in numbers after re-encountering newly injected tumor cells, followed by the subsequent drop in the peripheral blood (Fig. 5C). Further analysis of the blood T cells revealed that they exhibited effector/memory phenotype (Fig. 5C). Indeed, we found that the mice previously treated with γ-TCRγδ-T cells were resistant to A549 tumor cell re-challenge (Fig. 4C), possibly attributed to the persistence of infused γ-TCRγδ-T cells. Collectively, these results demonstrate that γ-TCRγδ-T cells possess superior anti-tumor activity against NSCLC.

### Anti-CD19 scFv fused to TCRγδ was functional and exhibited anti-tumor activities

**Fig. 6:**
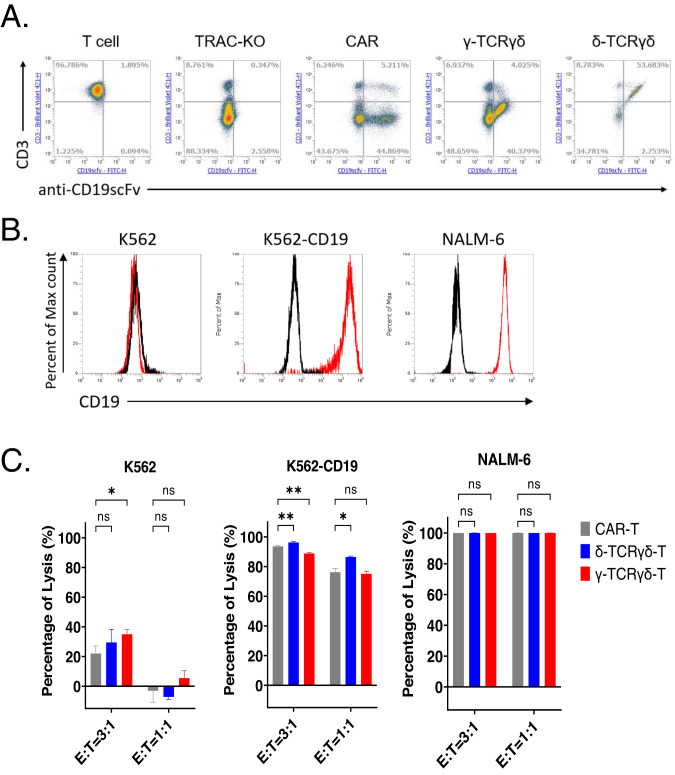
anti-CD19 scFv fused to TCRγδ is functional and exhibits antitumor activities. **A** Representative CAR, TCRγδ expression on transfected T cells at day 10 post transfection. The expression of CD3 and anti-CD19 scFv were assessed. **B** CD19 antigen expression in tumor cell lines used in this study was detected with flow cytometry. The histogram displays isotype-stained cells in black and CD19-stained cells in red. **C** Representative cytotoxic activity of CAR-T, δ-TCRγδ-T or γ-TCRγδ-T cells. The scFv expressing T cells at indicated E/T ratios were added to the tumor cell culture. Cytotoxicity was examined after overnight co-culturing by staining the tumor cells with Fixable viability dye and Annexin V. Percent of lysis was calculated by normalization to tumor only samples. All data are mean ± SEM. *p < 0.05, **p < 0.01, ***p < 0.001, ****p < 0.0001, ns: not significant.

The preceding findings regarding the surface expression of CD3 (Fig. 1C) and the in vivo efficacy (Fig. 3A) suggest that the synthetic δ-TCRγδ and γ-TCRγδ could exist in distinct configurations. To rule out the possibility that this applies to anti-PD-L1 scFv only, as well as to better benchmark our new receptor design with the well-studied CAR design, we re-constructed our TCR receptors with anti-CD19 scFv derived from clone FMC63 targeting human CD19. As expected, the flow cytometry analysis of anti-CD19 scFv TCR showed that the CD3 and scFv expression pattern was similar to that of anti-PD-L1 scFv TCR, indicating that the new receptor design of scFv fused to either δ-TCRγδ or γ-TCRγδ does differ in association with CD3 regardless of targeted scFv used (Fig. 6A). Then we used three tumor cell lines (K562, K562-CD19 and NALM-6) for the validation of the CD19 antigen specific killing of the engineered anti-CD19 scFv TCRγδ T cells (Fig. 6B). K562 cells do not express CD19 antigen while the K562-CD19 overexpress CD19 via lentivirus transduction. The NALM-6 is a B cell precursor leukemia cell line naturally expressing CD19. We performed overnight killing of the tumor cell lines with CAR-T, δ-TCRγδ-T or γ-TCRγδ-T. The results showed the three groups of engineered T cells can lyse the CD19 bearing tumor cell lines in a dose dependent manner while exerting minimal cytotoxic effect towards non-CD19-expressing K562 cell line (Fig. 6C). Taken together, the anti-CD19 scFv fused to TCRγδ replicated the in vitro cytotoxicity capability of killing target tumor cells in antigen specific manner of using anti-PD-L1 scFv. We believed that the new receptor design of scFv-TCRγδ could be applied to more scFv or other antigen binding moieties.

## Discussion

CAR-T therapy has achieved great success in the fight against hematopoietic malignancies, however, its application in the treatment of solid tumor continues to meet with major challenges. Firstly, it is very difficult to identify the ideal tumor specific targets, and owing to the heterogeneity of solid tumor, targeting one antigen is unlikely to eliminate all the tumor cells [10, 11]. Secondly, unlike liquid tumors, extensive infiltration of CAR-T and TCR-T cells into solid tumors, especially parenchyma, is hampered by many physical barriers [34]. Thirdly, it is still unclear how to sustain the survival and function of adoptive transferred T cells inside the tumor microenvironment. Research has shown that tumor infiltrated lymphocytes (TILs) tend to be hypofunctional due to the hypoxic conditions and upregulation of immune inhibitory molecules, such as PD-1 and CTLA-4, as well as suppression from the myeloid-derived suppressor cells (MDSCs) and regulatory T cells (Tregs) [35–37]. Therefore, an ideal cell therapy product targeting solid tumors may overcome at least some, if not all, of these hurdles.

Great efforts have been made in recent years to battle these challenges. For example, the combination of CAR-T and the blockade of PD1/PD-L1 has been shown to improve the mesothelin targeting CAR-T therapy in solid tumor [12]. Similarly, it has been reported that CAR-T engineered to secrete checkpoint inhibitor antibodies or CAR-T expressing chimeric PD1/CD28 which switches inhibiting signal to costimulatory signal could improve anti-tumor efficacy in solid tumor settings [38–42]. Interestingly, PD-L1 is abundantly expressed on many tumors under inflammatory conditions, serving as a tumor associated antigen. Indeed, in recent years, robust anti-solid tumor efficacy in xenograft models has been recorded by targeting PD-L1, with either PD-L1 CAR or dominant-negative form of PD1 [28, 29]. Based on these studies, we aimed to explore whether PD-L1 can be efficiently targeted by the TCR-T-based platform to mitigate the PD-1/PD-L1 mediated inhibition of TIL and to lyse tumor cells, ultimately eliminating solid tumors.

For this purpose, we constructed a new type of chimeric TCR by combining the advantages of both CAR-T and TCR-T to target PD-L1. The anti-PDL1 scFv was derived from Socazolimab, an anti-PDL1 antibody discovered by Sorrento Therapeutics and in development by Sorrento Therapeutics’ Licensing Partner, Lee’s Pharmaceutical and are now under Phase 3 study in China. We chose TCRγδ as the prototype to build the synthetic T cell receptor for several reasons. Firstly, the engagement of TCRγδ does not rely on additional co-stimulating signals to induce a strong proliferative response as compared to TCRαβ [43]. Secondly, since the endogenous TCRα chain is disrupted by gene editing occurring at the *TRAC* locus, a synthetic TCR using the TCRα chain could possibly pair with the endogenous TCRβ chain to form non-functional receptors. Therefore, the use of TCRγδ for constructing synthetic receptors could avoid the possibility of mismatching with endogenous TCRβ chain.

Our study revealed that anti-PD-L1 scFv fused to the TCRγδ heterodimer could efficiently target PD-L1-expressing tumor cells with a similar potency to anti-PD-L1 CAR in both in vitro and in vivo solid tumor models (Figs. 2, 3). We found that even in xenograft model, such as melanoma where CAR-T failed to function at all even with substantial T cell expansion, δ-TCRγδ-T and γ-TCRγδ-T cells could completely suppress the tumor growth (Fig. 3B), suggesting that the novel synthetic TCR could target a wider range of solid tumors than conventional CAR.

Furthermore, we discovered that when anti-PD-L1 scFv was fused to the γ-TCRγδ heterodimer, the resultant receptor demonstrated even stronger antitumor activities than δ-TCRγδ and exhibited greater expansion capacity (Fig. 4). A preclinical study showed that T cell antigen coupler (TAC), a chimeric receptor co-opts the TCR, engineered T cells outperformed CAR-T with faster tumor infiltration in the context of solid tumors [17]. In a solid tumor-bearing mouse model, we found that γ-TCRγδ-T infiltrated into tumor site and exhibited robust proliferative ability (Fig. 5A, B). γ-TCRγδ-T cells also demonstrated excellent persistency as evidenced by the re-challenge study (Fig. 5C). Interestingly, γ-TCRγδ and δ-TCRγδ exhibits distinct association with CD3 subunits, which could at least partially explain the observed difference in tumor killing and T cell expansion capacity. For a typical TCR, the antigen recognition and binding always involve two heterodimeric chains, either αβ or γδ, as well as the associated CD3 subunits. In the case of δ-TCRγδ where anti-PD-L1 scFv was tagged onto the TCRδ chain, the TCR-CD3 complex was readily detected on the cell surface by flow cytometry (Fig. 1C). In contrast, when scFv was fused to the TCRγ to form γ-TCRγδ, the CD3 expression was much lower at the cell surface, implying that the fusion of scFv to different chains could have distinct effect on the structure or conformation of the entire receptor complex, especially regarding the association with CD3 subunits [33]. Given the robust antitumor activity of γ-TCRγδ, the constant region of TCRδ appears to serve merely as an accessory chain within the synthetic TCRγδ heterodimer. Nevertheless, the exact biological significance of TCRδ chain in our synthetic anti-PD-L1 scFv GDT and the signaling transduction of γ-TCRγδ warrant further exploration.

It is worth noting that PD-L1 is extensively expressed in many types of tumors, as well as in normal cells. We noticed that the A549 cells with a slightly higher PD-L1 expression level than the A549 PD-L1 KO cells could be effectively killed by either γ-TCRγδ-T or δ-TCRγδ-T (Fig. 2A), implying possible on-target off-tumor toxicity. Hence, to alleviate potential systemic toxicity and to improve the safety profile of the novel anti-PD-L1 scFv TCR platform, it is advisable to integrate a suicide gene into the construct. In order to eliminate the possibility of our newly designed TCR being applicable specifically to anti-PD-L1 scFv and to perform a more comprehensive comparison between our new receptor design and the extensively studied CAR design, we rebuilt our TCR with anti-CD19 scFv sourced from clone FMC63 that targets human CD19. The in vitro tumor killing capability from CAR-T, δ-TCRγδ-T, and γ-TCRγδ-T after tumor killing exhibited a similar pattern to the results obtained from T cells engineered with anti-PD-L1 scFv. Therefore, it can be inferred that the fusion of anti-CD19 scFv to TCRγδ could mimic the in vitro cytotoxicity of anti-PD-L1 scFv by targeting and killing tumor cells in an antigen-specific manner. We anticipate that the scFv-TCRγδ receptor design could be extended to other scFv or antigen-binding moieties.

In summary, we have developed a new design of PD-L1-targeting γδ-based TCR-T that exerts anti-tumor function similarly to or even better than traditional CAR-T. The novel synthetic receptors γ-TCRγδ targeting PD-L1 demonstrated superior antitumor activities in both in vitro and in vivo solid tumor models, such as NSCLC and melanoma, supporting the strategy of inhibiting the PD1/PD-L1 axis to treat solid tumors. We also validated the same design with CD19 target with in vitro cytotoxicity assays. We believe that the innovative scFv-γ-TCRγδ could represent a new platform for cancer immunotherapy that awaits thorough investigation in future studies.

## Materials and methods

### Generation of plasmid and donor DNA

We synthesized all the fragments listed in (Supplementary Table 1) and cloned them into the pAAV-TRAC backbone, which contains the homology arms of *TRAC* locus flanking the synthetic promoter pJeT and the SV40 polyA. Using the plasmid as the template, the Donor DNA was made by PCR amplifying the fragments between the homology arms with PrimeSTAR HS DNA Polymerase (Takara Bio). The primer sequences are (Forward: ATCACGAGCAGCTGGTTTCT, Reverse: GACCTCATGTCTAGCACAGTTTTG). The PCR products were then purified using NucleoBond AX500 columns (Macherey-Nagel) according to the manufacturer’s protocol.

### gRNA, Cas9 protein and RNP formation

CRISPR-Cas9 crRNA and tracrRNA were purchased from IDT. To form a functional gRNA duplex, crRNA (CAGGGUUCUGGAUAUCUGUGUUUUAGAGCUAUGCU) and tracrRNA (Cat: 1072534, from IDT) were mixed at molar ratio 1:1 and heated at 95 °C for 5 min, followed by cooling down at room temperature (RT) for 20 min. Then the Cas9 protein (made in house) was added to the gRNA duplex at 1:2 molar ratio and incubated at 4 °C for 30 min. The TRAC ribonucleoprotein (RNP) was then used for gene editing experiments.

### Cell lines

Tumor cell line A549, SK-MEL-5, MDA-MB-231, K562 and NALM-6 were purchased from ATCC. A549 PD-L1 KO cell line was purchased from Abcam (ab267054). K562-CD19 cells were transduced with CD19 lentivirus in house and further selected and sorted. All tumor cell lines were transduced with Fluc-F2A-GFP-IRES-puro lentivirus and further selected and sorted. All cells were cultured in the medium according to ATCC’s handling information. All culture mediums were supplemented with 10% FBS, 2 nM L-glutamine (Invitrogen), 10 U/ml Penicillin and 10 ug/ml Streptomycin (MP Biomedicals). Typically, cells were split when reaching 70–80% confluency. All cell lines were mycoplasma free.

### T cell isolation and culture

Healthy donor LeukoPak was purchased from Hemocare. Peripheral blood mononuclear cells (PBMCs) were isolated by density gradient centrifugation and frozen in CryoStor CS10 (Sigma-Aldrich). On day 0, T cells were isolated from frozen PBMCs, activated with Dynabeads Human T-expander CD3/CD28 (Thermo Fisher Scientific) and cultured in CTS OpTmizer T cell expansion medium (Thermo Fisher Scientific) supplemented with 5% human serum, and 300 U/ml of IL-2. On day 3, the Dynabeads were removed with magnets, and cells were continued to culture for one day. Each ensuing cellular or animal experiment was performed using T cells from at least two different donors. In all figure legends, the data were obtained using T cells from one donor only with technical triplicates.

### Gene editing

Activated T cells on day 4 were washed with PBS and transfected by electroporation of TRAC RNP and donor DNA using the Neon Transfection System (Thermo Fisher Scientific). Typically, 4 × 10^6^ T Cells were resuspended in R buffer and mixed 250 pmol RNP complex and 20 ug donor DNA product to a total volume of 100 μl. After transfection, cells were cultured at the density of 1 × 10^6^ cells/ml. The medium was changed every 2–3 days and the cells were maintained at 1–2 × 10^6^/ml. At different time points as indicated in the text, T cells were examined for the phenotype, assessed function, or subjected to adoptive transfer.

### Flow cytometry

For detecting the anti-PD-L1 scFv, a PD-L1-Fc Fusion protein was used (Sino Biological). For detecting the anti-CD19 scFv, a monoclonal anti-FMC63 antibody was used (Acro Biosystems). For T cell phenotype characterization, the following antibodies were used: mouse anti-human CD3 (clone: UCHT1), TCRαβ (clone: IP26), CD4 (clone: RPA-T4), CD8 (clone: RPA-T8), CD45RA (clone: HI100), CD62L (clone: DREG-56), CCR7 (clone: G043H7) from BioLegend. To evaluate cytotoxicity, Live/Dead Fixable Yellow (Thermo Fisher Scientific) and Annexin V (BioLegend) were used to stain the effector and tumor cells in the co-culture. To examine the degranulation, CD107a (BioLegend) and Granzyme B (BD) were used in the assay. FACS data were acquired on Attune (Thermo Fisher Scientific) and analyzed with Attune software.

### Degranulation assay

To measure the cytolytic function of T cells, the scFv expressing T cells were incubated with target cells at 1:1 ratio. T cells without target cell stimulation were served as controls. CD107a antibody was added to the culture when starting the coculture. Cells were incubated at 37 °C in 5% CO_2_ for 2 h. Brefeldin A (BioLegend) was then added to the culture and the coculture was continued for 3 h. The cells were harvested and subjected to surface staining for 15 min. cells were then fixed, permeabilized with Cytofix/Cytoperm Fixation/Permeabilization solution kit (BD) according to the manufacture’s protocol and analyzed on Attune.

### Cytotoxicity assay

Cytotoxicity assay was performed using A549, A549 PD-L1 KO, SK-MEL-5, MDA-MB-231, K562, K562-CD19, NALM-6 cell lines expressing GFP-Firefly Luciferase (GFP-Fluc) or RFP-Firefly Luciferase (RFP-Fluc) as target cells. Briefly, 1 × 10^5^ target cells were plated per well in 96-well round bottom plate (Corning). The scFv expressing T cells were served as effector cells and added at indicated effector to target (E/T) ratio. After 4 h or overnight co-culture, the plates were stained with Live/Dead Fixable Yellow (Thermo Fisher Scientific), followed by washing with Binding buffer and stained with Annexin V (BioLegend). Samples were then analyzed by FACS. The cytotoxicity was calculated as the percentage of Annexin V^+^ and Live/Dead^+^ cells gated on the target cells.

In some experiments, evaluation of cytolytic activity was done by Agilent xCELLligence RTCA system. One day prior to the addition of effector cells, 6 × 10^3^ A549 tumor cells were seeded in a 96-well E-Plate, effector cells were added at the indicated E/T ratio, the measurement of cell growth (cell adhesion) were taken every 15 min for a period of up to 250 h.

### Cytokine release

To measure the secreted cytokines, 1 × 10^5^ scFv expressing T cells were cocultured with 1 × 10^5^ tumor cells in 96-well plate at 37 °C overnight. Supernatant were collected and cytokines were measured using IFN-γ and GM-CSF ELISA kits (Thermo Fisher Scientific) and read by Cytation5 imaging reader. The OD values were calculated, and the data were plotted using GEN5 software.

### Immunohistochemistry

Tumor tissues were removed and fixed with the 10% formalin solution. Fixed tissue blocks were paraffin-embedded and sectioned. Slides were then deparaffinized, rehydrated, and treated for antigen retrieval. The slides were then blocked with normal goat serum for 20 min at RT and incubated with primary antibody against human CD45 at 4 °C overnight. Slides were further incubated with biotinylated secondary antibody (Vector Laboratories) for 30 min at room temperature. The slides were then incubated with the peroxidase detection system, VECTASTAIN Elite ABC Reagent, for 30 min at RT. Following this step, the slides were incubated with the peroxidase substrate solution (Vector Laboratories) for 10 min at RT, until desired intensity was obtained. The slides were counterstained with hematoxylin QS (Vector Laboratories), dehydrated, mounted, and visualized by Olympus CX33 Digital Microscope.

### In vivo xenograft model

Seven-week-old female NSG mice were purchased from JAX and housed at the Sorrento Vivarium. For the NSCLC xenograft model, 3 × 10^6^ A549 tumor cells mixed with Matrigel were administered subcutaneously on the dorsal flank of the mouse. Mice with established tumors were randomly divided into different groups when xenograft tumor reached about 200 mm^3^. TRAC-KO, CAR-T, δ-TCRγδ-T, or γ-TCRγδ-T cells were injected intravenously via tail vein (i.v.) to tumor bearing mice. For re-challenge study, 57 days after γ-TCRγδ-T cells treatment (30 days after tumor complete regression), mice were re-challenged with 3 × 10^6^ A549 tumor cells and followed for 3 weeks. For melanoma xenograft model, 4 × 10^6^ SK-MEL-5 tumor cells mixed with Matrigel were administered subcutaneously on the dorsal flank of the mouse. The tumor grafted mice were divided into groups randomly when tumor reached about 200 mm^3^. TRAC-KO, δ-TCRγδ-T, or γ-TCRγδ-T cells were injected i.v. to tumor bearing mice. For the breast cancer xenograft model, 1 × 10^6^ MDA-MB-231 tumor cells mixed with Matrigel were administered subcutaneously on the dorsal flank of each mouse. The tumor grafted mice were divided into groups randomly when tumor reached about 200 mm^3^. TRAC-KO, δ-TCRγδ-T, or γ-TCRγδ-T cells were injected i.v. to tumor bearing mice. Tumor volume and body weight were measured, and adoptive transferred T cells were monitored by analyzing the peripheral blood samples taken at indicated time points. Mice were sacrificed when the tumor volume measured was over 2000 mm^3^ and the data was excluded from the result. Animal experiments were conducted under protocols approved by the Institutional Animal Care and Use Committee at Sorrento Therapeutics.

### Statistics

Data are shown as the mean ± SEM in the figures. Statistical analysis was performed using analysis of variance (ANOVA) test. Statistical significances were considered as *P < 0.05, **P < 0.01), ***P < 0.001, and ****P < 0.0001. All statistical analyses were performed using GraphPad Prism software.

## Supplementary information

Supplementary Information

Supplementary Table 1

Supplementary Figure 1

Supplementary Figure 2

## Data Availability

All data generated or analyzed during this study are included in this published article and supplementary information files.
