# Supplementary material for: Antibody-based binding domain fused to TCRγ chain facilitates T cell cytotoxicity for potent anti-tumor response
Source: Oncogenesis. 2023 Jun 22;12(1):33. doi: 10.1038/s41389-023-00480-4 (PMC10287668; doi:10.1038/s41389-023-00480-4)
Supplement: Supplementary file 1 — Supplementary Information [file 41389_2023_480_MOESM1_ESM.pdf]

Supplementary Information

Supplementary Table 1

Construct illustrations and sequences of fragments flanked by pJeT promoter and SV40 PolyA. The table lists the constructs and amino acid sequences of the synthetic TCR used in this study. The sequence underlined is the signal peptide.

Supplementary Figure 1

Flow cytometry result of degranulation marker CD107a and Granzyme B expression for Figure 2B.

Supplementary Figure 2

Xenograft result of another donor related to Figure 3A. A549 WT-bearing NSG mice were treated with 1e7 CAR-T (n=10), δ-TCRγδ T (n=10) or γ-TCRγδ T cells (n=10), as well as equal cell number of TRAC-KO T cells as a control group (n=10). Tumor size was measured at indicated time points and analyzed over a 40-day period (left panel). Peripheral blood was drawn at indicated time points and numbers of $CD45^{+}$ cells were calculated (middle panel). Kaplan-Meier survival curve (right panel) of A549 WT-bearing NSG mice was shown. Statistical significance was calculated using the log-rank Mantel-Cox test (n=10 per cohort).

All data are mean±SEM. *p<0.05, **p<0.01, *** p<0.001, ****p<0.0001, ns: not significant.
