# Supplementary material for: Antibody-based binding domain fused to TCRγ chain facilitates T cell cytotoxicity for potent anti-tumor response
Source: Oncogenesis. 2023 Jun 22;12(1):33. doi: 10.1038/s41389-023-00480-4 (PMC10287668; doi:10.1038/s41389-023-00480-4)
Supplement: Supplementary file 2 — Supplementary Table 1 [file 41389_2023_480_MOESM2_ESM.pdf]

Supplementary Table 1

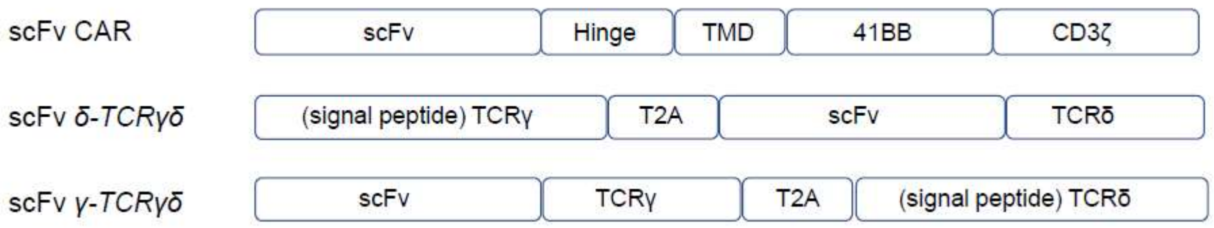

(signal peptide) TCRγ	MLSLLHTSTLAVLGALCVYGFPPIKTDVITMDPKDNCSKDANDTLLLQLTNTSAYYMYLLLLLKSVVYFAIITCCLLRRTAFCCNGEKS
(signal peptide) TCRδ	MQRISSLIHLSLFWAGVMSTDFEVKTDSTDHVKPKETENTKQPSKSCHKPKAIVHTEKVNMMSLTVLGLRMLFAKTVAVNFLLTAKLFFL
T2A	GSGATNFSLLKQAGDVEENPGP
CD8α/CD28 Hinge and TMD	AKPTTTPAPRPPTPAPTIASQPLSLRPEACRPAAGGAVHTRGLDFAPRKIEVMYPPPYLDNEKSNGTIIHVKGKHLCPSPLFPGPSKPFWVLVVVGGVLACYSLLVTVAFIIFWV
CD8α Hinge and TMD	TTTPAPRPPTPAPTIASQPLSLRPEACRPAAGGAVHTRGLDFACDIYIWAPLAGTCGVLLLSLVITLYC
41BB	KRGRKKLLYIFKQPFMRPVQTTQEEDGCSCRFPEEEEGGCEL
CD3ζ	RVKFSRSADAPAYQQGQNQLYNELNLGRREEYDVLDKRRGRDPEMGGKPRRKNPQEGLYNELQKDKMAEAYSEIGMKGERRRGKGHDGLYQGLSTATKDTYDALHMQALPPR
PD-L1 scFv	MEWSWVFLFFLSVTTGVHSEVQLVESGAEVKKPGSSVKVSCKASGGTFSSYAISWVRQAPGQGLEWMGGIIPIFGTANYAQKFQGRVTITADESTSTAYMELSSLRSEDTAVYYCARAPYYYYYMDVWGQGTTVTVSSGGGGSGGGGSGGGGSQSALTQPASVSGSLGQSVTISCTGSSSDVGSYNLVSWYQQHPGKAPNLMIYDVSKRSGVSNRFSGSKSGNTASLTISGLQAEDEADYYCSSYTGISTVVFGGGTKLTVL
CD19 scFv	MSVPTQVLGLLLLWLTDARCDIQMTQTTSSLSASLGDRVTISCRASQDISKYLNWYQQKPDGTVKLLIYHTSRLHSGVPSRFSGSGSGTDYSLTISNLEQEDIATYFCQQGNTLPYTFGGGTKLEITGGGGSGGGGSGGGGSEVKLQESGPGLVAPSQSLSVTCTVSGVSLPDYGVSWIRQPPRKGLEWLGVIWGSETTYYNSALKSRLTIIKDNSKSQVFLKMNSLQTDDTAIYYCAKHYYYGGSYAMDYWGQGTSVTVSS
