# Supplementary figures and images for: Antibody-based binding domain fused to TCRγ chain facilitates T cell cytotoxicity for potent anti-tumor response
Source: Oncogenesis. 2023 Jun 22;12(1):33. doi: 10.1038/s41389-023-00480-4 (PMC10287668; doi:10.1038/s41389-023-00480-4)

# Supplementary Figure 1

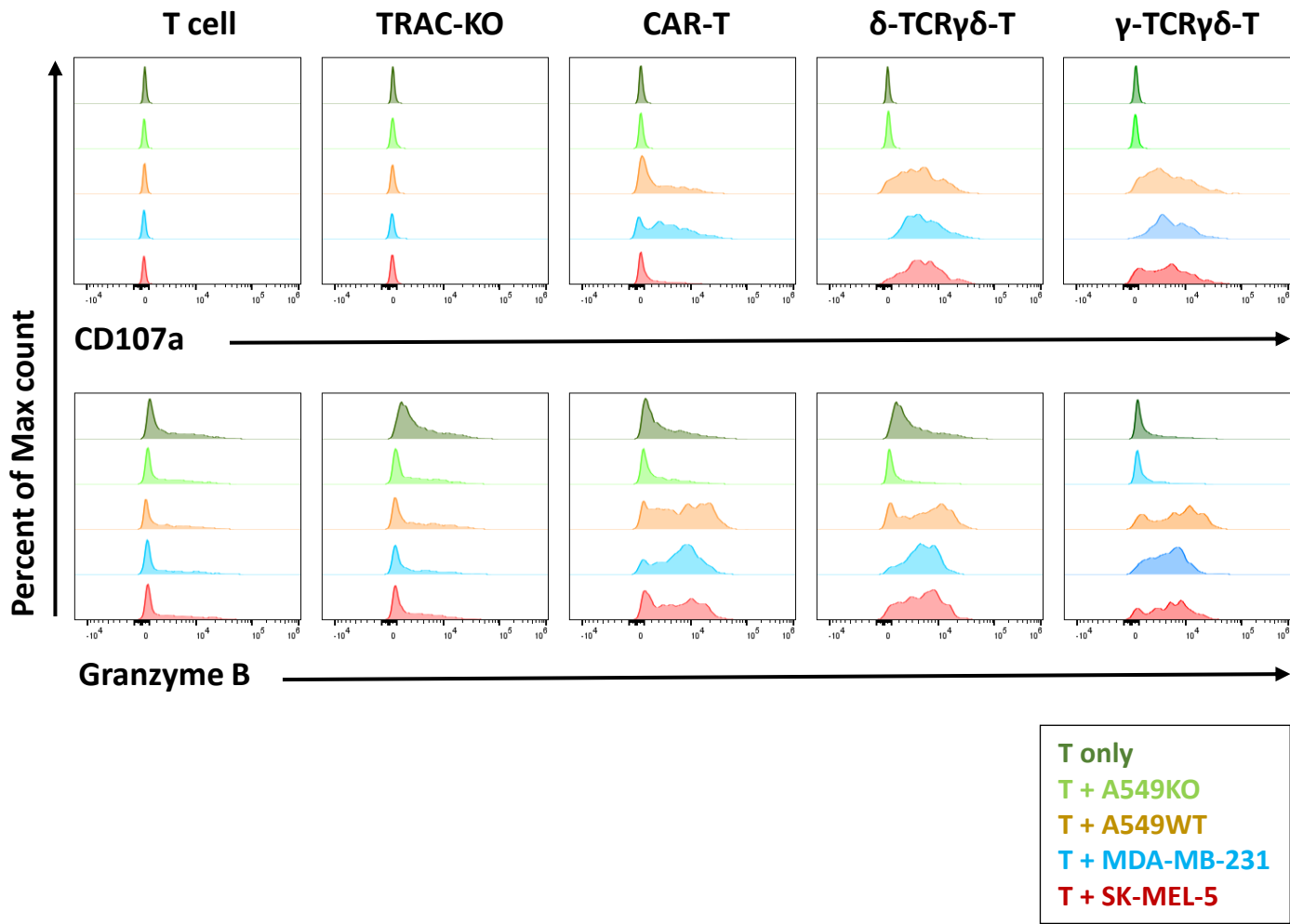

Supplement: Supplementary file 3 — Supplementary Figure 1 [file 41389_2023_480_MOESM3_ESM.pdf]

# Supplementary Figure 2

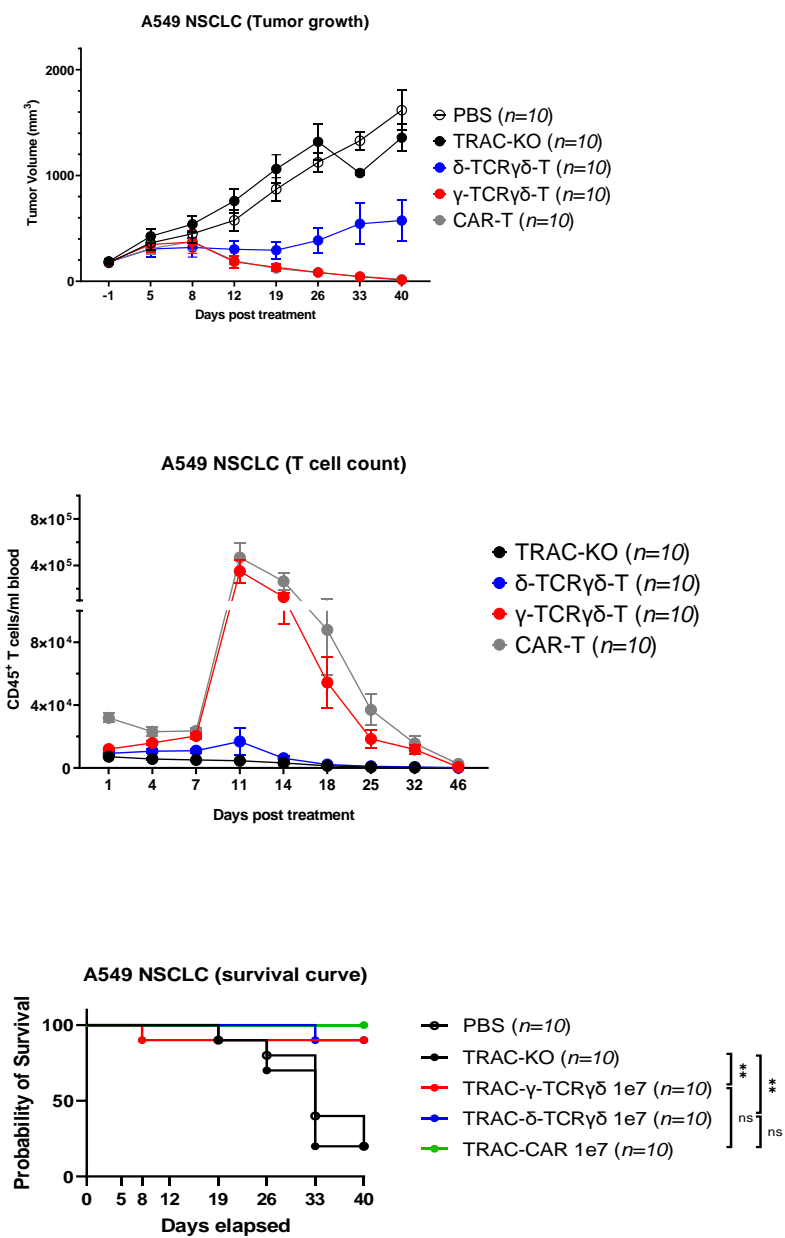

Supplement: Supplementary file 4 — Supplementary Figure 2 [file 41389_2023_480_MOESM4_ESM.pdf]
